# Genotypic and phenotypic characterization of enteroaggregative *Escherichia coli* (EAEC) isolates from diarrheic children: An unresolved diagnostic paradigm exists

**DOI:** 10.22038/ijbms.2020.42119.9959

**Published:** 2020-07

**Authors:** Haiffa Helalat, Seyedeh Elham Rezatofighi, Mohammad Roayaei Ardakani, Luis Fernando Dos Santos, Mahdi Askari Badouei

**Affiliations:** 1 Department of Biology, Faculty of Science, Shahid Chamran University of Ahvaz, Ahvaz, Iran; 2 Adolfo Lutz Institute, Centere of Bacteriology, National Reference Laboratory for *E. coli* enteric infections and HUS. São Paulo, Brazil; 3 Department of Pathobiology, Faculty of Veterinary Medicine, Ferdowsi University of Mashhad, Mashhad, Iran

**Keywords:** Aggregative adherence, Biofilm, Diagnosis, Diarrhea, Enteroaggregative-Escherichia coli, Virulence genes

## Abstract

**Objective(s):**

The enteroaggregative *Escherichia coli* (EAEC) has been one of the most intriguing emerging bacterial pathogens in children that occur both in developing countries and the industrial world. Although various phenotypic and genotypic based protocols have been suggested for diagnosis of EAEC, they are not conclusive or practical to be used in most clinical laboratories.

**Materials and Methods:**

In this study, we analyzed and compared 36 typical EAEC strains (*aggR*-positive) by various genotypic and phenotypic methods.

**Results:**

Briefly, pCVD432 was detected in all of isolates along with aggR, then it was followed by other virulence genes including *app, astA, aggA*, and *pet* genes in 32 (88.8%), 21 (58.3%), 9 (25%), and 2 (5.5%) isolates, respectively. Biofilm was formed by 34 (94.4%) isolates, while only 26 (72.2%) isolates showed an aggregative adherence pattern to HEp-2 cells.

**Conclusion:**

The genetic and phenotypic features of EAEC were highly inconsistent, which may have considerable diagnostic implications. The variations in the virulence genes, phenotypic characteristics, and genetic profiles among the EAEC isolates again emphasized the genetic heterogeneity of this emerging pathotype. Biofilm formation may be an important phenotypic virulence property of this pathotype, especially in strains with the *aggR*-pCVD432-*aap-astA* profile.

## Introduction

Enteroaggregative *Escherichia coli* (EAEC), a pathotype of diarrheagenic *E. coli *(DEC), affects children in both developing and developed countries worldwide (1). EAEC has been classically shown to cause chronic intestinal inflammation and persistent diarrhea in children leading to malnutrition and growth retardation (2, 3). It is also the second cause of travelers’ diarrhea after enterotoxigenic *E. coli* (ETEC) strains (3). This pathotype is recognized by its distinctive aggregative adherence (AA) pattern to HEp-2 cells characterized by a “stacked-brick” appearance (1, 4). The strains of the EAEC pathotype have a heterogenic profile due to the presence of several putative determinants. Although, in some cases EAEC has been isolated from asymptomatic children, it caused several outbreaks and fatal cases of infections especially where children were suffering from malnutrition and poor hygiene (5). The relationship between a specific genotype and its pathogenicity in humans has not as yet been understood, probably due to the tremendous genetic diversity, heterogeneity of virulence factors, and chronic nature of infection (5, 6). The pathogenesis of EAEC strains is not discovered in detail, but adherence to the intestinal mucosa by aggregative adherent fimbriae (AAF) and biofilm formation on gastric mucosa is the first critical step. Next, the bacteria release cytotoxins and several enterotoxins that contribute to secretory diarrhea, inflammatory response, and mucosal cytotoxicity (7, 8). 

One of the most important virulence carrying repertoire of EAEC isolates is a 60- to 65-MDa plasmid that leads to the aggregative adherence (AA) phenotype and is named pAA (6). This plasmid harbors many putative factors associated with EAEC pathogenesis including AAFs, the transcriptional activator AggR, antiaggregation protein (dispersin), the heat-stable toxin-1 (EAST-1), *aatA* (which corresponds to CVD432 fragment), and plasmid encoded toxin (PET) (2, 6, 9). The use of plasmid encoded probe known as “CVD432” or the AA probe has been proposed for the molecular identification of the EAEC isolates (4, 6, 8). Due to the important role of *aggR*, Nataro proposed that EAEC strains with *aggR* regulon be known as “typical EAEC,” (tEAEC) (5). Investigations regarding the clinical and epidemiological properties of diarrheal disease should consider detection of tEAEC (9-11). AAFs are responsible for the AA phenotype (10, 12). Five variants of AAF, AAF/I–AAF/V, are described so far (9, 13, 14); however, some strains with AA phenotype do not harbor any of the discovered fimbriae suggesting that additional unknown AAF variants may exist (4, 15, 16). AFFs can lead to the formation of biofilm, an important pathogenic trait of EAEC (9, 14, 16). Treatment of EAEC infection can be difficult as mucosal biofilm creates a barrier that prevents the penetration of antibiotics and host antimicrobial factors (17). One of the other EAEC virulence factors that are present in a minority of the strains is the pAA plasmid-encoded toxin (PET), which cleaves the membrane cytoskeletal protein, spectrin. EAST-1 is another virulence factor associated with the EAEC isolates that is encoded by the *astA* gene (5, 10), although it is not restricted to EAEC (9). EAST-1 reduces the absorption of water and electrolytes by the enterocytes of the intestine (15).

Although comparing to other DEC, investigations on EAEC are scarce many studies have suggested that EAEC is much more prevalent than the other DEC in the industrialized and developing countries as reported in UK (18), USA (19), Brazil (20), and India (21). Similar to other countries, very few studies considered EAEC detection in Iran, but a few reports showed the highest prevalence among DEC (22). In our recent study in Southwest Iran (Khouzestan province), we observed that EAEC constituted more than 60% of DEC isolates in diarrheic children (23); therefore, in the present research we aimed to compare phenotypic and genotypic features of EAEC for the first time in Iran.

## Materials and Methods

***Bacterial strains***

In this study, 36 non-duplicate EAEC isolates that were collected in our previous investigation (23) were analyzed. All the EAEC strains were derived from 208 stool samples of children with diarrhea in Ahvaz city, Iran, during 2015–2016. All of the investigated isolates were identified as *E. coli* by biochemical tests and PCR amplification of the *uidA* gene and confirmed as typical EAEC using *aggR* gene-specific PCR.

***Biofilm formation assay***

The strains were grown in Luria-Bertani (LB) medium (Merck, Germany) at 37 ^°^C. Two hundred microliters of Dulbecco’s modified Eagle’s medium (DMEM) (GIBCO, Scotland) containing 0.45% glucose was added to 96-well flat-bottom microtiter polystyrene plates (SPL; South Korea), and then, 5 μl of each overnight culture of isolates was inoculated to each well. The samples were incubated at 37 ^°^C, and after 18 hr, the culture was removed, and the biofilm was stained with 0.5% crystal violet for 5 min; the wells washed with 1×PBS, and air dried. Two hundred microliters of 95% ethanol was added to each well, and the absorbance was measured using a plate reader (Bio-Rad; USA) at 570 nm. The isolates were classified into three categories as high, weak, and no biofilm producers.* The* isolates with OD_570_ readings lower than the mean plus two standard deviations of the *E. coli* DH5α (OD_570_≤0.38) were considered negative biofilm producers. The strains with OD_570_>0.38<0.76 and OD_570_≥0.76 readings were categorized as weak and strong biofilm producers, respectively. EAEC strain 042 and *E. coli* DH5α served as positive and negative controls for the strong biofilm producer, and the non-biofilm producer, respectively. All the assays were carried out in triplicate (10). 

***Adhesion assay***

HEp-2 cells were grown in RPMI1640 (GIBCO, Scotland) with 10% fetal bovine serum (FBS) (GIBCO, Scotland), penicillin (100 U/mL), and streptomycin (100 µg/mL) at 37 ^°^C under 5% CO_2_ in a 24-well tissue culture plate to produce a monolayer with 50% –70% confluence. Then, the culture medium was replaced with fresh RPMI1640 with 2% FBS, and 1% D-mannose without antibiotics. The EAEC isolates were cultured in a nutrient broth overnight at 37 ^°^C, and then, 35 µl of each sample adjusted to OD_600_ = 2 was inoculated to each well and incubated at 37 °C for 3 hr. Next, the cells were washed twice with PBS, fixed with 100% methanol, and stained with 10% Giemsa for 5 min. The AA patterns were examined under 40 magnification using an inverted microscope (Olympus; Japan) (6). All the assays were carried out in triplicate.

***Invasion assay***

The assay was performed as previously described (6). The first step of the test was done as described for the adhesion assay with the difference that 3 hr after the inoculation of the isolates into the HEp-2 cells, the cells were washed three times with PBS, and the cell culture medium containing 250 µg/ml amikacin was added to each well to kill the extracellular bacteria (all isolates were sensitive to amikacin). Next, the monolayer cells were incubated for an additional 1 hr, washed with PBS, and then, lysed with 1% Triton X-100 in deionized water for 5 min. The culture media containing lysed cells were removed, diluted, and cultured on nutrient agar media, and the number of CFU was calculated after 24 hr to quantify the number of intracellular bacteria and measure the invasion. The percentage of the original inoculum isolate resisting treatment with amikacin was measured to express the invasion levels. The assays were performed in duplicate and at least three times in independent experiments (6). 

***Detection of virulence genes***

Boiling method was used to prepare the DNA template for PCR. In brief, an individual colony was inoculated to 1 mL nutrient broth and incubated at 37 ^°^C for 6 hr. Then, the culture was centrifuged, and the supernatant was removed. The bacterial plate was suspended in 300 µl distilled water and boiled for 10 min. Next, the sample was centrifuged at 11000 g for 10 min, and the supernatant was harvested and stored at 20 ^°^C until use. All isolates were examined by PCR for pCVD432, *aggA*, *aafA*, *aap*, *pet*, and *astA* genes. The reaction conditions were previously described (8, 16, 24-26). The details of the primers are presented in Table 1.

***Enterobacterial repetitive intergenic consensus (ERIC)-PCR fingerprinting***

The extracted DNA of the isolates was analyzed by ERIC-PCR reaction, as previously described (27, 28). The reaction mixture of 25 μl volume contained 12.5 μl master mix with a final concentration of 2 mM MgCl_2_ (Amplicon, Denmark), 0.4 μM of each primer (Table 1), and 25 ng template DNA. The amplifications were carried out with a Bio-Rad (USA) thermal cycler (1 cycle at 94 ^°^C for 7 min; 35 cycles at 94 ^°^C for 30 sec, 50 ^°^C for 1 min, and 72 ^°^C for 3 min; and 1 cycle at 72 ^°^C for 15 min). A data matrix was compiled, to assess the relatedness between the isolates, based on the presence or absence of a band with the score of 1 or 0, respectively. The SIMQUAL program in NTSYS-pc, version 2.02e, was used to calculate Jaccard’s similarity coefficients between the isolates. The data were used for UPGMA cluster analyzing using the SAHN NTSYS program. 

***Statistical analysis***

The data were analyzed using the Fisher’s exact and χ2 tests. A *P*-value equal to or less than 0.05 was considered significant.

## Results

***Genotypic characterization of EAEC isolates***

In the previous study, EAEC strains were detected by confirming the presence of the *aggR* gene; as a result, all strains were positive for *aggR*. All strains were also positive for the pCVD432 fragment (which corresponds to pAA plasmid). Thirty two (88.8%) isolates harbored the *aap* gene, which encodes dispersin, and revealed perfect concordance between pCVD432-*aggR* positivity and the presence of the *aap* gene. The *aap* gene was the most commonly identified virulence gene followed by *astA*, which was identified in 21 (58.3%) isolates. The frequencies of virulence genes among the EAEC isolates are shown in Table 2. Fimbrial encoding gene of *aggA* was found in 9 (25%) isolates, while none of the strains had the *aafA* gene. Finally, *pet* gene was identified in 2 (5.5%) isolates. Two isolates harbored only the pCVD432-*aggR*, but not the other virulence genes investigated; however, they showed the AA phenotype. Statistical analysis revealed that there was no significant relationship between virulence factors. Several different profiles of the virulence gene combinations were found among the isolates (Table 3). The most prevalent combination was pCVD432-*aggR*-*aap-astA,* which was found in 13 (36.1%) strains.

***Phenotypic characterization of EAEC isolates***

The results of adherence, invasive, and biofilm assays are presented in Table 2. Adherence to the cells is a critical first step for bacterial pathogenesis (6). All isolates were analyzed for adhering to HEp-2 cells. The AA pattern was observed in 26 (72.2%) isolates; however, in spite of the presence of pCVD432-*aggR*, which defined tEAEC strains, 10 (27.7%) isolates were not adherent and did not reveal the AA pattern on the HEp-2 cells, which is still used to define the EAEC pathotype. The ability to form biofilm using EAEC isolates was semi-quantitatively assessed using the microliter plate assay. Accordingly, 30 (83.3%) and 4 (11.1%) isolates were high and weak biofilm producers and only 2 (5.5%) isolates were unable to produce biofilm. One of the factors that could be involved in the bacterial pathogenesis is the ability to invade cells. Several studies revealed that some EAEC strains possessed this ability (9, 29). In the present study, 7 (19.4%) and 3 (8.3%) isolates showed low (<0.05% of the original inoculum) and high (>0.05% of the original inoculum) invasion rates, respectively and the rest of them were unable to invade the cells. 

***ERIC-PCR***

**Figure 1:**
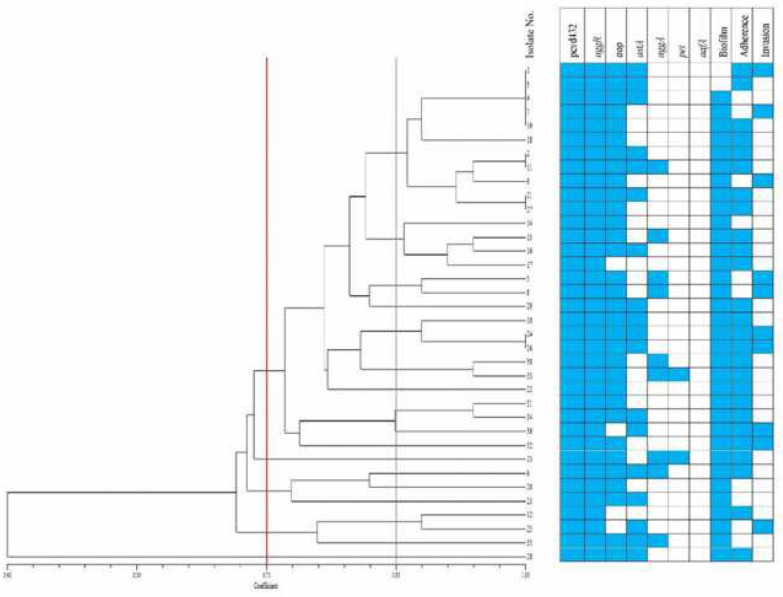
Enterobacterial repetitive intergenic consensus (ERIC) dendrogram and characteristics of enteroaggregative *Escherichia coli* (EAEC) isolates causing diarrhea in children

The ERIC-PCR banding patterns yielded 5 to 25 bands encompassing in size from 100 bp to 5000 bp. A dendrogram was constructed based on analysis of DNA bands using NTSYS-pc V 2.02e. UPGMA clustering differentiated all the isolates into 5 clusters and 17 sub-clusters at a coefficient of 0.71 and 0.85, respectively. The genetic diversity and relatedness of the *E. coli *strains were shown in Figure 1. In the same cluster, *E. coli *isolates are the most genetically related to ones and are more homogenous; while according to the Figure 1 these isolates have different phenotypic profiles. However, some isolates in different clusters or sub clusters have similar phenotypic profiles. *E. coli *isolates were placed in the same cluster but isolated from different persons or times suggested that these strains might be circulating isolates in the environment. 

## Discussion

Although some studies investigated the association of virulence genes and EAEC pathogenesis, little is known about the major attributes in the virulence of this pathotype. Currently, three methods are used for identification of the EAEC pathotype, including phenotypic cell culture assays, molecular detection, or combined methods (30). The studied isolates in our research were previously identified as EAEC based on the detection of the flagship *aggR* gene. The pCVD432 fragment was also detected in all isolates; therefore, these isolates were considered as tEAEC. Approximately 72% of the isolates revealed the AA pattern, and the rest were unable to adhere to the HEp-2 cells. Presumably, only the presence of pCVD432 and *aggR* regulon is not sufficient to yield the “stacked bricks” pattern. Some isolates with the “stacked-brick” mode are negative for both of these fragments and are known as atypical EAEC (31). In fact, as a result of discrepancies in diagnosis of EAEC, we cannot clearly define the highly pathogenic EAEC clones and efficiently track related epidemics. These results suggest that relying on the observation of “stacked-brick” pattern may not be sufficient to detect some possible diarrheagenic strains within this pathotype. Although, it should be noted that the extended incubation time may increase the sensitivity of the adherence assay. 

In this study, the virulence gene content of EAEC was quite diverse, which shows the mosaic nature of EAEC genome, possibly the result of extensive horizontal gene transfer (HGT) and recombination (32, 33). One of the genes that has been frequently found in the EAEC is *aap* (9). In our study, more than 86% of the isolates harbored this gene with the combination of *aggR* and pCVD432. Strains carrying the *aap* gene can overcome the mucus layer that is produced in response to bacterial infection by the production of dispersin (34).

Our results also revealed that 25% of the isolates harbored AAF/I, but AAF/II was not found in any of the isolates. The frequency of these fimbriae among the EAEC isolates is highly variable in different studies; for example, AAP/I frequency has been reported to be from 0% (35) to 63% (36). It is suggested that the prevalence of the operon AAF/II is low, and our results corroborate these findings (12, 37). Some researchers have suggested that the presence of AAF/II is a marker for pathogenic EAEC strains (37, 38). Obviously, the high heterogeneity of this operon prevents its detection by the conventional methods (12), and therefore, its diagnostic value needs to be evaluated. Overall, a comprehensive study that described all AAF variants is not yet introduced (15). Comparative genomics on large EAEC collections of different origins seems to be necessary to clarify the virulence attributes of this versatile pathogen and its important virulence plasmid.

In the past, the *astA* gene was considered a character of the EAEC pathotype, but later, this gene was found among the other pathotypes and commensal isolates (39); therefore, it was concluded that the EAST toxin is not sufficient to cause diarrhea unless combined with other virulence factors (34). However, few DEC strains were found that harbor no virulence factors other than EAST (39). *astA* was found in more than half of the isolates and approximately 78% and 89% of them showed an AA pattern and *aap* gene, respectively. Presumably, a combination of these characters with each other can contribute to the pathogenesis of the isolates. 

One of the important pathogenicity factors of EAEC is the biofilm formation which occurs mainly in the colon and to a lesser extent in the small intestine. It is believed that many persistent and chronic bacterial infections are closely associated with biofilm formation (37). The evaluation of biofilm formation has been introduced as a possible method for the screening of pathogenic EAEC isolates (11). In our study, more than 94% of the isolates were able to form a biofilm and at least 85% of them were detected as high biofilm producers. In different studies, the ability of the EAEC isolates to form biofilm varied and a prevalence of 50%–100% for biofilm formation has been reported (37, 40). Such variations confirm that this test is not similarly applicable as a reliable diagnostic option. The similarity in the biofilm formation in our study may indicate the presence of a main cluster and its sub-clusters in the studied area. We need more details of the genetic lineages by multi locus sequence typing (MLST) analysis to confirm this because ERIC-typing did not verify such hypothesis. The ERIC profile revealed that the isolates were divided into five clusters; however, similar isolates harbored different virulence gene profiles or phenotypic characteristics (Figure 1). Although, we have successfully used ERIC-typing with good discriminatory power for STEC strains (41), it might be a less efficient tool for EAEC clonal analysis. This phenomenon may also be explained by the pAA plasmid transfer to a diverse pool of gut *E. coli* with different genetic backgrounds. 

**Table 1:** Names and sequences of primers used for ERIC-PCR and PCR amplification of virulence genes

Primer name	Sequence (5'→3')		References
	Forward	Reverse	
*aggR*	ACGCAGAGTTGCCTGATAAAG	AATACAGAATCGTCAGCATCAGC	
pCVD432	CTGGCGAAAGACTGTATCAT	AATGTATAGAAATCCGCTGTT	29
*app*	CTTGGGTATCAGCCTGAATG	AACCCATTCGGTTAGAGCAC	
*pet*	ACTGGCGGACTCATTGCTGT	GCGTTTTTCCGTTCCCTATT	
*astA*	TGCCATCAACACAGTATATCCG	ACGGCTTTGTAGTCCTTCCAT	
*aggA*	TTAGTCTTCTATCTAGGG	AAATTAATTCCGGCATGG	
*aafA*	TGCGATTGCTACTTTATTAT	ATTGACCGTGATTGGCTTCC	
ERIC	ATGTAAGCTCCTGGGGATTCAC	AAGTAAGTGACTGGGGTGAGCG	

**Table 2:** Frequency of two virulence-related marker combinations among enteroaggregative *Escherichia coli* (EAEC) strains isolated from children with diarrhea (N=36)

	pCVD432 N (%)	*aggR * N (%)	*aggA * N (%)		*astA * N (%)	*aafA * N (%)	*aap * N (%)	*pet * N (%)	Biofilm N (%)	Adherence N (%)	Invasion N (%)
*pcvd432*	36 (100)		
*aggR*	36 (100)	36 (100)								
*aggA*	9 (25)	9 (25)	9 (25)							
*astA*	21 (58.3)	21 (58.3)	3 (8.3)		21 (58.3)					
*aafA*	0 (0)	0 (0)	0 (0)		0 (0)	0 (0)				
*aap*	32 (88.8)	32 (88.8)	9 (25)		16 (44.4)	0 (0)	32 (88.8)			
*pet*	2 (5.5)	2 (5.5)	2 (5.5)		0 (0)	0 (0)	2 (5.5)	2 (5.5)		
Biofilm	34 (94.4)	34 (94.4)	9 (25)		16 (44.4)	0 (0)	30 (83.3)	2 (5.5)	34 (94.4)	
Adherence	26 (72.2)	26 (72.2)	6 (16.6)		14 (38.8)	0 (0)	22 (61.1)	2 (5.5)	24 (66.6)	26 (72.2)
Invasion	10 (27.7)	10 (27.7)	2 (5.5)		5 (13.8)	0 (0)	8 (22.2)	0 (0)	8 (22.2)	5 (13.8)	10 (27.7)

**Table 3:** The frequency of different virulence gene combinations among enteroaggregative *Escherichia coli* (EAEC) strains isolated from children with diarrhea

Genetic profiles	EAEC isolates n (%)
pCVD432-*aggR-aap-astA*	13 (36.1)
pCVD432-*aggR-aap*	10 (27.7)
pCVD432-*aggR-astA*	5 (13.9)
pCVD432-*aggR-aap-aggA*	4 (11.1)
pCVD432-*aggR-aap-aggA-astA*	3 (8.3)
pCVD432-*aggR*	2 (5.5)
pCVD432-*aggR-aap-aggA-pet*	2 (5.5)

## Conclusion

The variation in the virulence genes and phenotypic traits of EAEC isolates reassured the genetic heterogeneity of this pathotype and the subsequent challenges it causes in clinical laboratories. Additionally, unlike many well recognized DEC strains, some important typing schemes like serotyping have been less promising for EAEC due to vast genetic diversity and presence of many unknown antigenic variants (32). It seems that the current status of EAEC diagnosis is not equivalent to its clinical relevance, especially in countries that children are suffering from the triads of malnutrition, poor hygiene, and subsequent infections. Although, biofilm formation and AA pattern in cell culture have been the most important diagnostic criteria of this pathotype, we still need to develop more practical and reliable diagnostic options for screening of EAEC in clinical settings. 
